# Author Correction: Sculpting nanoparticle dynamics for single-bacteria-level screening and direct binding-efficiency measurement

**DOI:** 10.1038/s41467-019-09171-4

**Published:** 2019-03-12

**Authors:** Y. Z. Shi, S. Xiong, Y. Zhang, L. K. Chin, Y.–Y. Chen, J. B. Zhang, T. H. Zhang, W. Ser, A. Larsson, S. H. Lim, J. H. Wu, T. N. Chen, Z. C. Yang, Y. L. Hao, B. Liedberg, P. H. Yap, K. Wang, D. P. Tsai, C.-W. Qiu, A. Q. Liu

**Affiliations:** 1 School of Mechanical Engineering, Xi’an Jiaotong University, 710049 Xi’an, China; 2 School of Electrical and Electronic Engineering, Nanyang Technological University, Singapore, 639798 Singapore; 3 School of Mechanical and Aerospace Engineering, Nanyang Technological University, Singapore, 639798 Singapore; 4 Department of Electrical and Computer Engineering, National University of Singapore, Singapore, 117583 Singapore; 5 School of Biological Sciences, Nanyang Technological University, Singapore, 639798 Singapore; 6 National Key Laboratory of Science and Technology on Micro/Nano Fabrication, Institute of Microelectronics, Peking University, 100871 Beijing, China; 7 Centre for Biomimetic Sensor Science, School of Materials Science and Engineering, Nanyang Technological University, Singapore, 639798 Singapore; 8 Lee Kong Chian School of Medicine, Nanyang Technological University, Singapore, 308232 Singapore; 9 College of Biomedical Engineering, Taipei Medical University, Taipei, 11031 Taiwan; 10 Nanyang Environment & Water Research Institute, Nanyang Technological University, Singapore, 637141 Singapore; 11 Department of Physics, National Taiwan University, Taipei, 10617 Taiwan; 12 SZU-NUS Collaborative Innovation Center for Optoelectronic Science and Technology, Shenzhen University, 518060 Shenzhen, China

Correction to: *Nature Communications*; 10.1038/s41467-018-03156-5; published online 26 February 2018

The original version of this Article omitted the author Kuan Wang, who is from the ‘College of Biomedical Engineering, Taipei Medical University, Taipei 11031, Taiwan’ and ‘Nanyang Environment & Water Research Institute, Nanyang Technological University, Singapore 637141, Singapore.’

Also, the author S.H. Lim was incorrectly given as L.S. Hoi and A. Larsson was incorrectly given as A. Larson.

The “Author contributions” was amended to reflect the authorship changes. It previously read ‘Y.Z.S., C.-W.Q., and A.Q.L. jointly conceived the idea. Y.Z.S., S.X., Y.Z., J.B.Z., W.S., J.H.W., T.N.C., Z.C.Y., Y.L.H., B.L., P.H.Y., D.P.T., and C.-W.Q. performed the numerical simulations and theoretical analysis. Y.Z.S., S.X., and L.K.C. did the fabrication and experiments of particle hopping, biomolecule binding and flow cytometry. A.L. and L.S.H. did the SPR experiments. S.X., Y.Z.S., Y.Z., C.-W.Q., Y.-Y.C., L.K.C., T.H.Z., and A.Q.L. prepared the manuscript. S.X., Y.Z., C.-W.Q., and A.Q.L. supervised and coordinated all the work. All authors commented on the manuscript.’ The correct version states ‘B.L., K. W., P.H.Y.’ instead of ‘B.L., P.H.Y.’ and ‘S.H.L.’ in place of ‘L.S.H.’

This has been corrected in both the PDF and HTML versions of the Article.

