# Supplementary material for: Sculpting nanoparticle dynamics for single-bacteria-level screening and direct binding-efficiency measurement
Source: Nat Commun. 2018 Feb 26;9:815. doi: 10.1038/s41467-018-03156-5 (PMC5827716; doi:10.1038/s41467-018-03156-5)
Supplement: Supplementary file 1 — Supplementary Information [file 41467_2018_3156_MOESM1_ESM.pdf]

SUPPLEMENTARY INFORMATION FOR

Sculpting Nanoparticle Dynamics for Single-bacteria-level Screening and Direct Binding-efficiency Measurement

Y. Z. Shi et al.

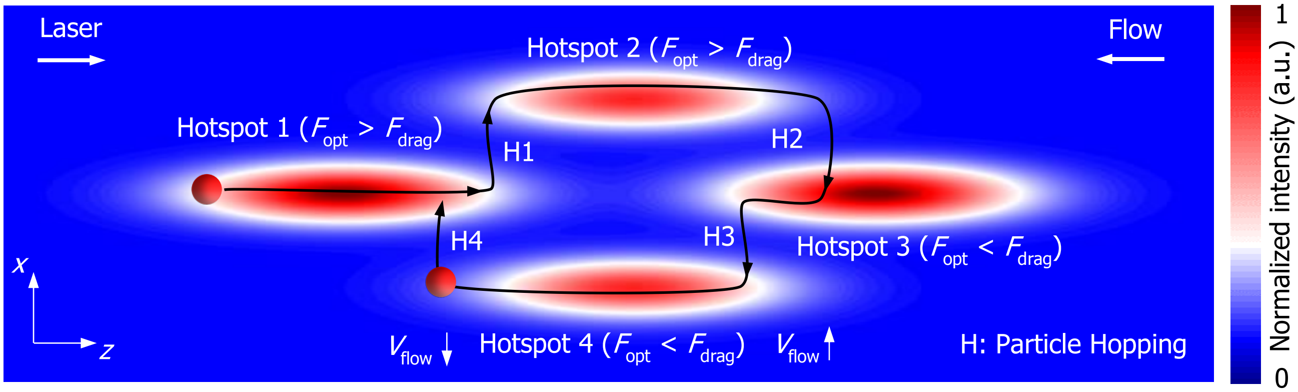

Supplementary Fig. 1 | Configuration of the 2D controllable optical hopping loop in an optofluidic lattice. Step 1: The particle is pushed to the right edge of hotspot 1 by the optical scattering force and then hops to hotspot 2. Step 2: The particle in hotspot 2 is pushed to the right edge by the optical scattering force, then hops to hotspot 3. In Step 1 and 2, optical scattering force is larger than the fluidic drag force. Step 3: By increasing the flow velocity, the drag force becomes larger than the optical force. The particle is driven to the left edge by the flow. Then, by changing the flow direction slightly downward, the particle hops to hotspot 4 instead of hopping back to hotspot 2. Step 4: Since the drag force is still larger than the optical force, the particle is driven to the left edge of hotspot 4. After that, the flow velocity is decreased while the flow is shifted back to horizontal direction. Finally, the particle hops to hotspot 1.

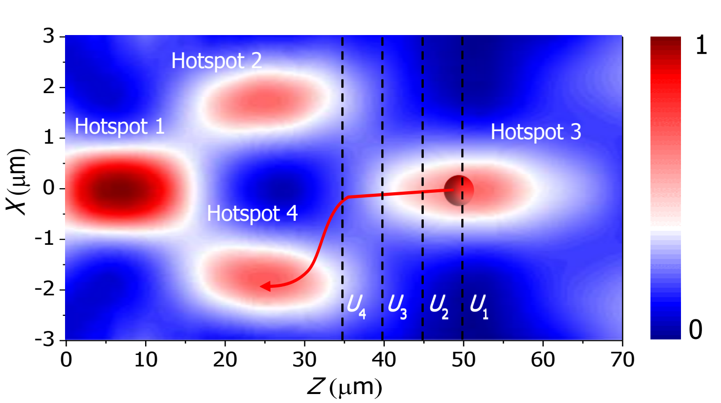
Hotspot 2
Hotspot 1
Hotspot 3
Hotspot 4
U_4 U_3 U_2 U_1
X (μm)
Z (μm)
1
0

(a)

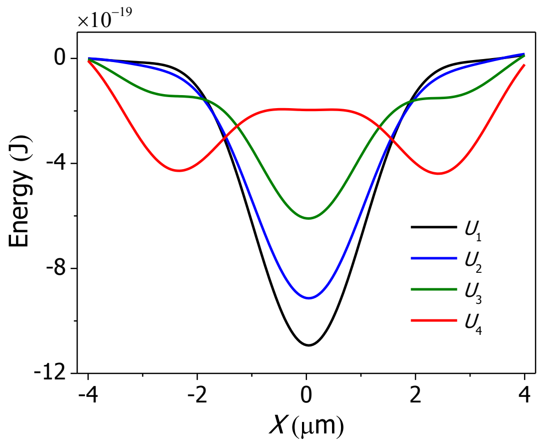
×10⁻¹⁹
Energy (J)
X (μm)
U_1
U_2
U_3
U_4

(b)

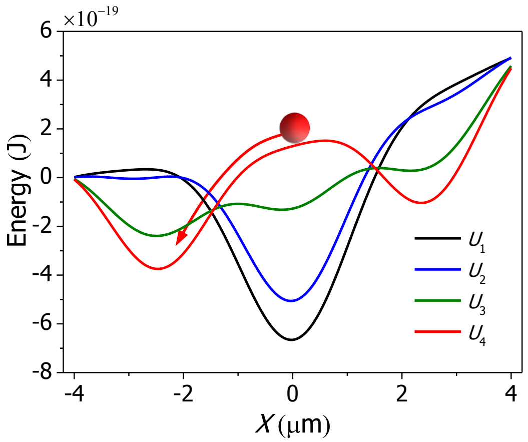
×10⁻¹⁹
Energy (J)
X (μm)
U_1
U_2
U_3
U_4

(c)

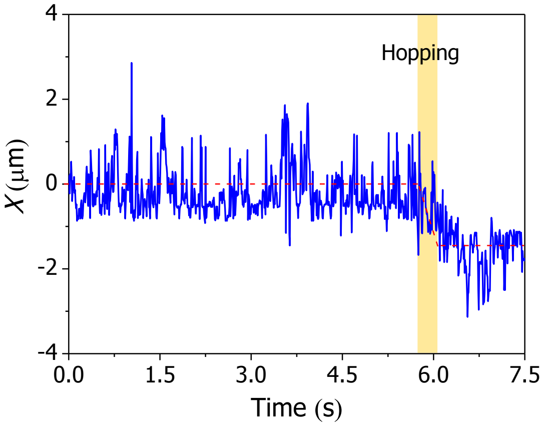
Hopping
X (μm)
Time (s)

(d)

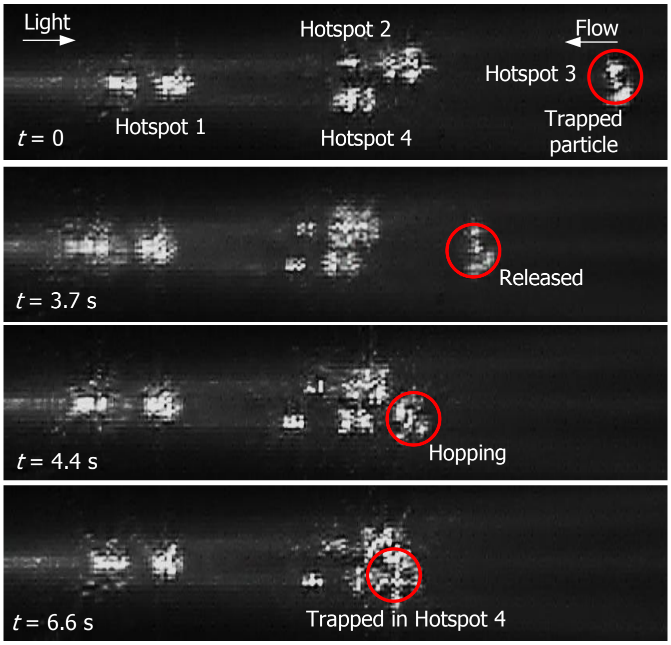
Light
Hotspot 2
Flow
Hotspot 3
Trapped particle
Hotspot 1
Hotspot 4
t = 0
Released
t = 3.7 s
Hopping
t = 4.4 s
Trapped in Hotspot 4
t = 6.6 s

(e)

Supplementary Fig. 2 | Particle hopping triggered by the drag force. (a) 1-μm particle (red) is driven by the drag force and hop from hotspot 3 to 4 subsequently through four potential wells (dashed lines) labelled with U_1 (z = 50 μm), U_2 (z = 45 μm), U_3 (z = 40 μm) and U_4 (z = 35 μm). The intensity pattern is rescaled for a better illustration. (b) Energy profile of U_1, U_2, U_3 and U_4 without lateral drag force in x−direction. (c) Energy profile of U_1, U_2, U_3 and U_4 with the lateral drag force in x−direction when the lateral flow velocity is 10 μm s^{-1}. The lateral force is generated when the velocity of upper sheath flow is slightly increased by approximately 20 μm s^{-1}, which causes the asymmetry of the hydrodynamic focusing. The lateral velocity should not be larger than 50 μm s^{-1}, or else, the particle will escape from hotspot 4 without being captured by hotspot 1. The drag force is estimated to be increased from 0.19 to 0.45 pN. (d) Particle trajectory during the hopping from hotspot 3 to 4. The oscillation of the particle is due to the Brownian motion. The particle changes its x-position after reaching U_4. (e) Experimental demonstration of the particle hopping triggered by the fluidic drag force. The particle was trapped in the hotspot 3 at t = 0, and was pushed away by the flow at t = 3.7 s. It hopped to the hotspot 4 at t = 4.4 s and was stably trapped at t = 6.6 s. See also the **Supplementary Movie 6**.

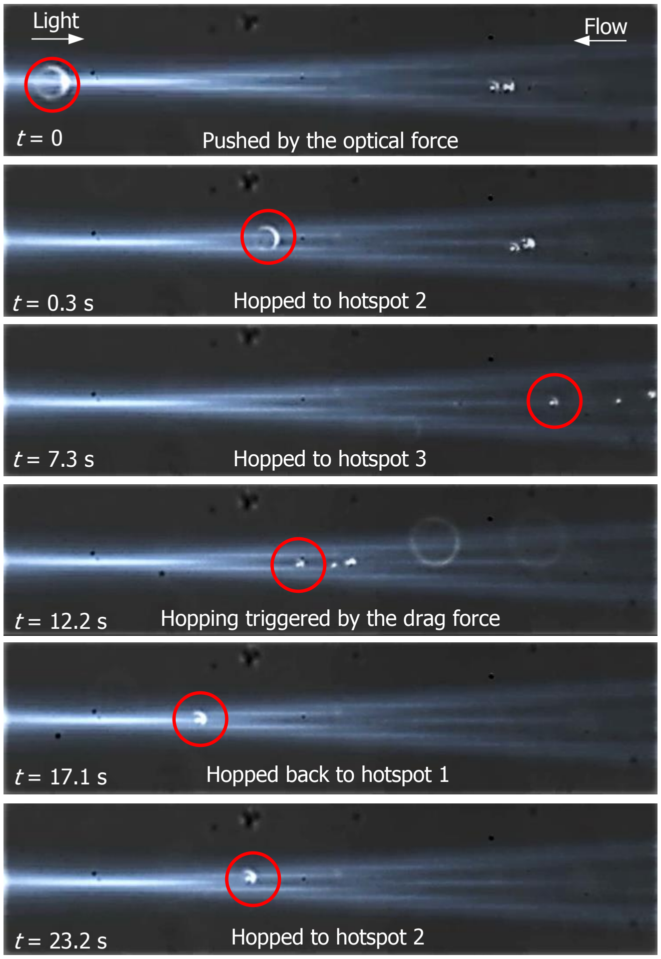

Supplementary Fig. 3 | Experimental demonstration of the controllable optical hopping loop in an optofluidic lattice. At t = 0, the particle was pushed to the right edge by the optical scattering force. It then hopped to hotspot 2, and after that to hotspot 3 at t = 7.3 s. To generate the hopping loop, the flow velocity was increased from 100 to 200 μm s^{-1} and a lateral velocity 20 μm s^{-1} was applied. The particle then hopped to hotspot 4 by the fluidic drag force at t = 12.2 s. It finally hopped back to hotspot 1 at t = 17.1 s. By decreasing the velocity to 100 μm/s, the particle hopped to hotspot 2 again. See also the **Supplementary Movie 7**.

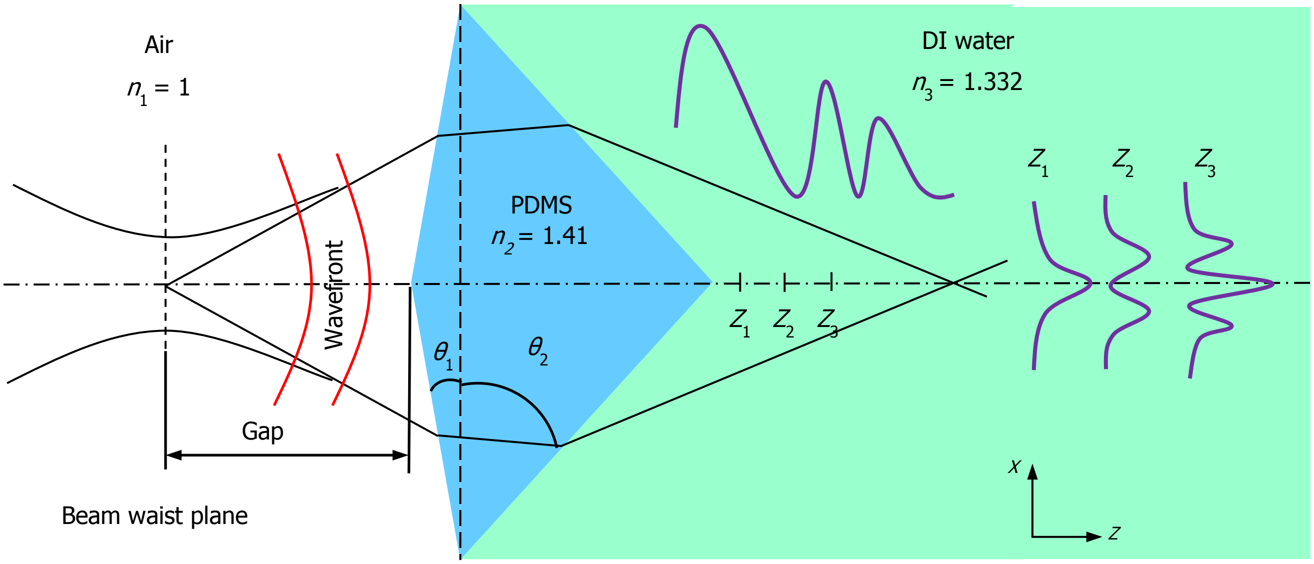

Supplementary Fig. 4 | 2D model of the micro-quadrangular lens. The micro-quadrangular is made of poly-dimethyl siloxane (PDMS) (refractive index n = 1.41). The open angles (θ_1 and θ_2) of the two triangles are 10.67 °and 55 °, respectively.

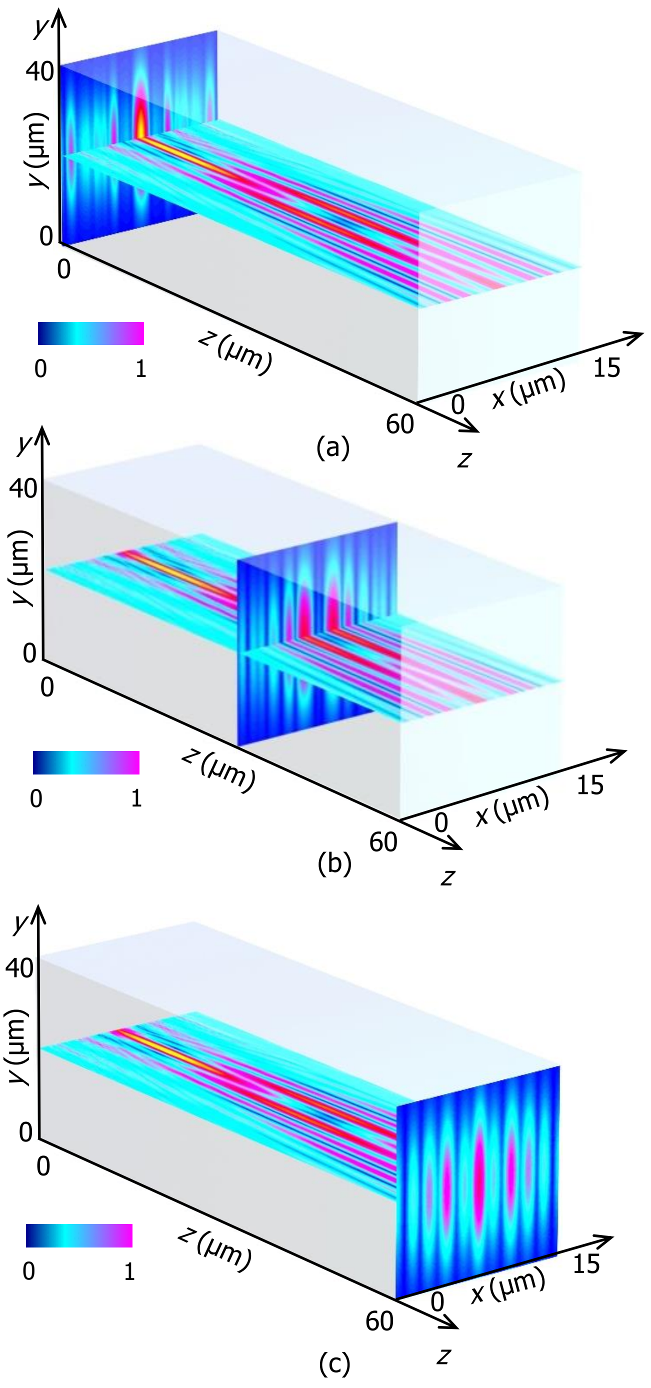

Supplementary Fig. 5 | 3D simulation of the discrete interference pattern. The discrete interference pattern with the cross section plots in z = (a) 0, (b) 30 μm and (c) 60 μm.

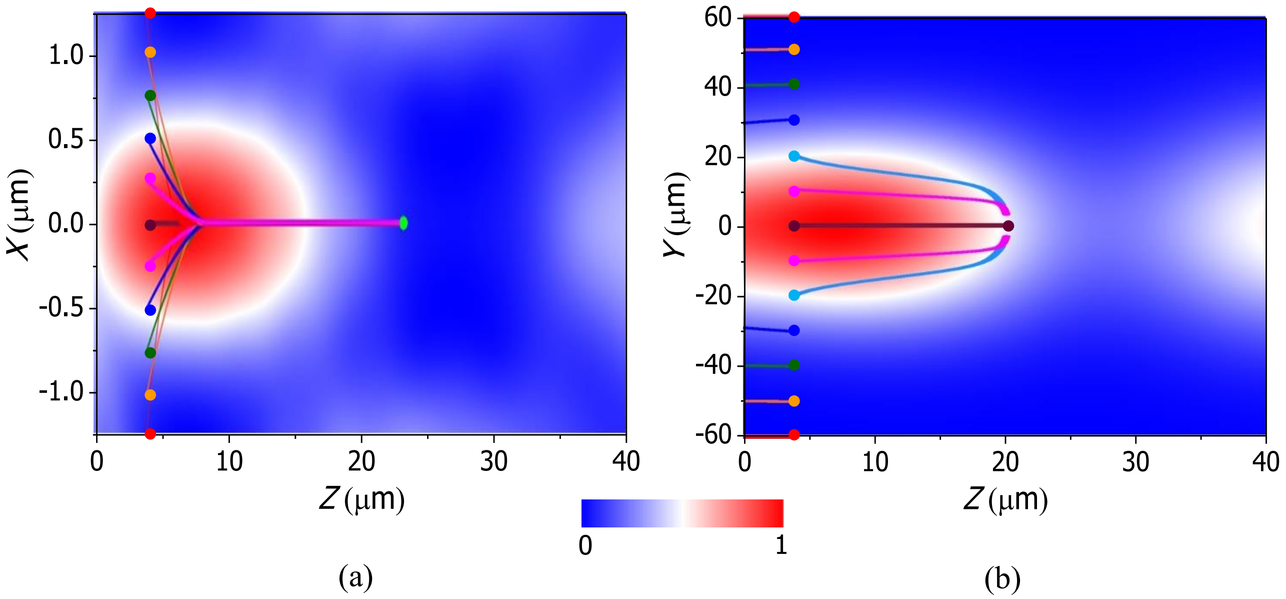

Supplementary Fig. 6 | Simulation of particle trajectories in (a) *x*−*z* and (b) *y*−*z* planes. The dots represent the starting points of the particles when $t = 0$. The trajectories ending time of the particles in (a) and (b) are 0.3 and 3 seconds, respectively. (a) In the *x*-*z* planes, the starting points of the particles are at $z = 5$ μm, which are on the left edge of hotspot 1. *x* is from -1.25 to 1.25 μm. After $t = 0.2$ s, all particles are attracted to the center line of the hotspot ($x = 0$) and pushed to the right edge of the hotspot. (b) In the *y*-*z* planes, the starting points of the particles are also at $z = 5$ μm. *y* is from -60 to 60 μm. The strong optical force drives all particles in the hotspot (-20 μm < *y* < 20 μm) to move towards the stable trapping point ($y = 0$ and $z = 20$ μm), whereby the optical force balances the fluidic drag force. While particles beyond this range (-20 μm < *y* < 20 μm) are flushed away by the drag force.

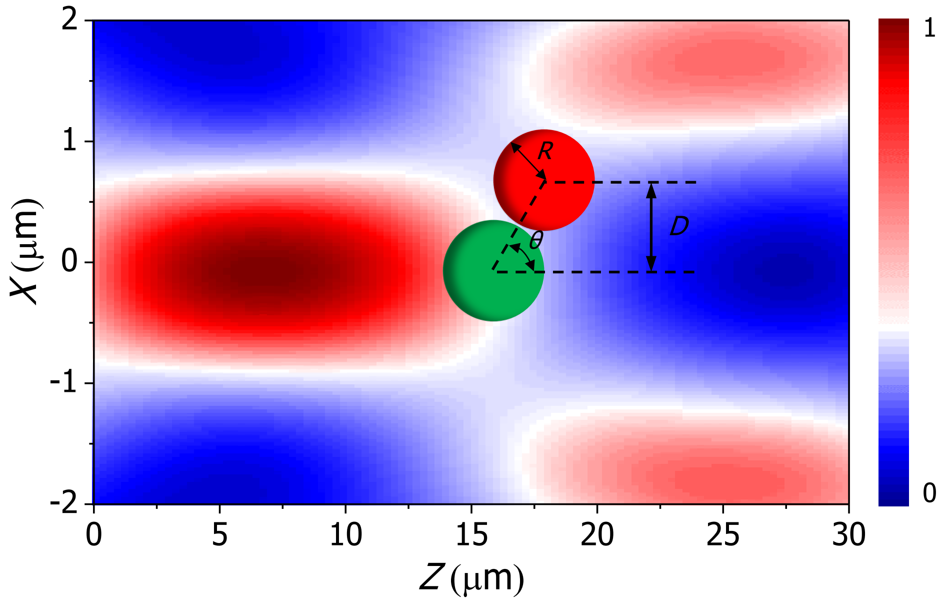

Supplementary Fig. 7 | Illustration of the contact angle and the vertical distance between the two particles after collision. The distance D depends on the particle radius R and contact angle θ. The contact angle is defined as the angle between the line connecting two mass centers of particles with z-direction.

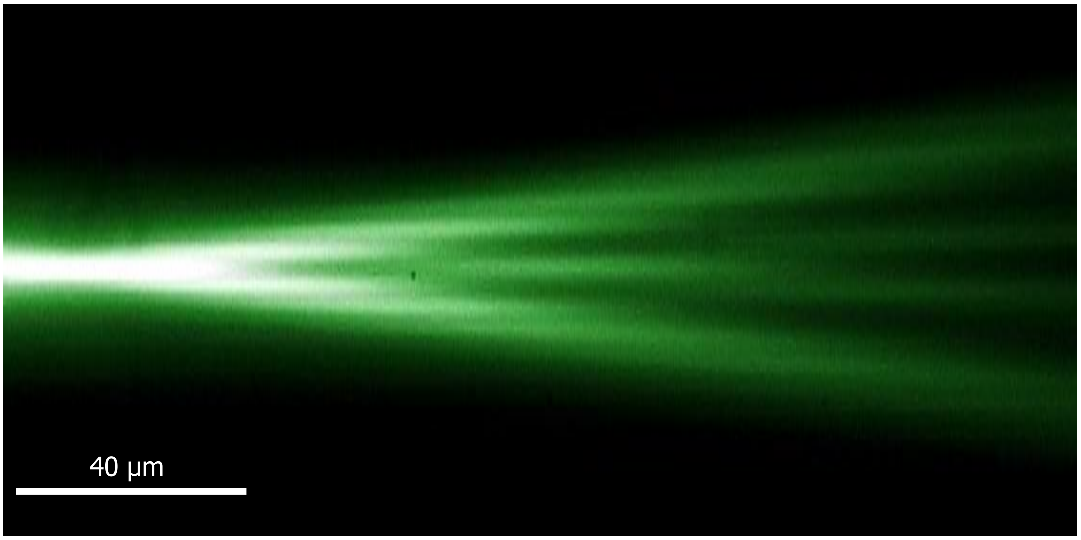

Supplementary Fig. 8 | Fluorescence image of the discrete interference pattern. The height of this image is stretched to 5 folds of the original one.

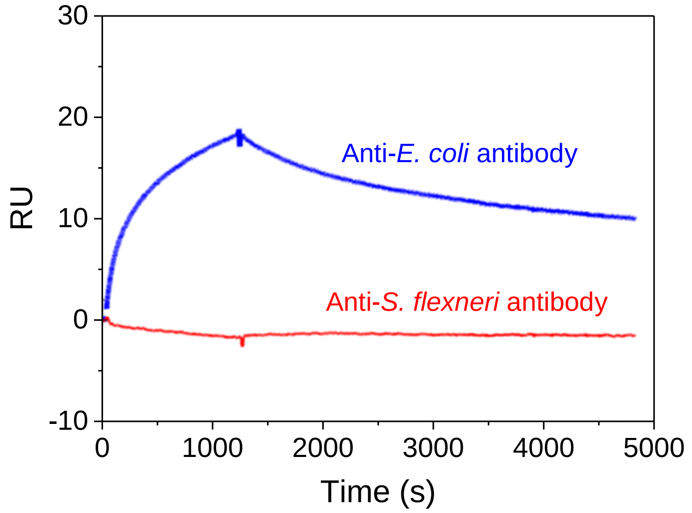

(a) *E. coli* cells

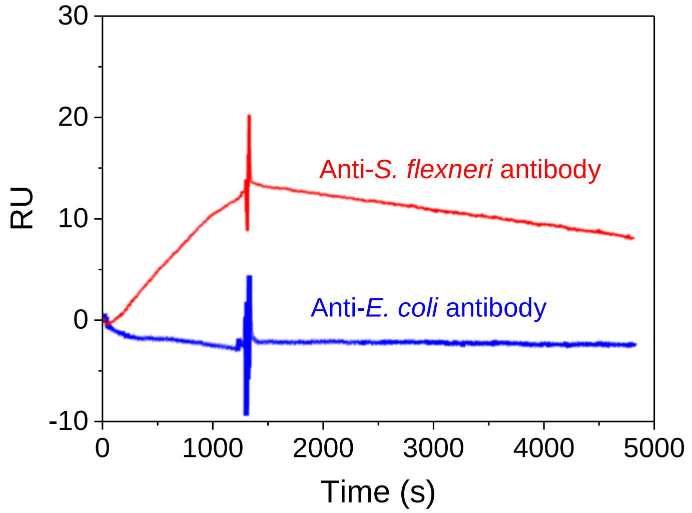

(b) *S. flexneri* cells

Supplementary Fig. 9 | SPR experiments for the binding of bacteria and antibodies. Anti-*E. coli* antibodies (blue) and anti-*Shigella* antibodies (red) were captured to the density of approximately 600 RU on flow cell 2 and 4 respectively; flow cell 1 and 3 was left blank to serve as a reference surface. PBS buffer blank or the analytes, (a) *E.coli* cells (4×10^7 cells ml^{-1}) and (b) *S. flexneri* cells (4×10^7 cells ml^{-1}) in PBS buffer were injected over the all flow cells at a flow rate of 1 μl min^{-1}, for a contact time of 1200 s and dissociate for 5400 s. Data were collected at a rate of 1 Hz, and at a temperature of 25 ℃. Sensorgrams were plotted with a common *y* scale to allow direct comparison of the different samples. When *E. coli* cells flew to the anti-*E. coli* antibodies (blue curve, cell 2), the RU signal increased with time while injecting, meaning there were binding between the bacteria and antibodies. When *E. coli* cells flowed to the anti-*S. flexneri* antibodies (red curve, cell 4), the RU signal remained in 0, meaning there were no binding. Similarly, *S. flexneri* cells bound only to anti-*S. flexneri* rather than anti-*E. coli* antibodies as shown in (b).The slopes of the off rate in the binding curves in (a) and (b) are very close, indicating the binding strength of the *E. coli* to anti-*E. coli* antibody and *S. flexneri* to anti-*S. flexneri* antibodies is similar.

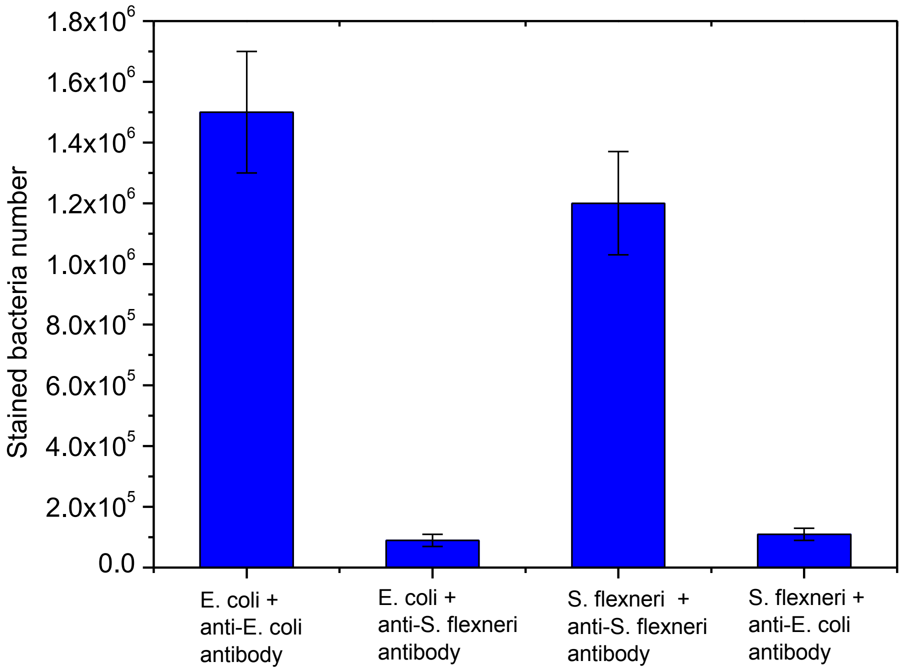

Supplementary Fig. 10 | Flow cytometry experiments for the binding of bacteria and antibodies. Anti-*E. coli* antibodies and anti- *S. flexneri* antibodies were conjugated with fluorescent dye (Alexa Fluor 568). *E.coli* cells (4×10^7 cells ml^{-1}) and *S. flexneri* cells (4×10^7 cells ml^{-1}) in PBS buffer were both incubated with dye-conjugated anti-*E. coli* antibodies and anti-*S. flexneri* antibodies, respectively. The samples were analyzed by flow cytometry after washing twice and diluted 10 times. The stained bacteria numbers were determined by fluorescence and side scattering intensity from 15 samples, with error bars indicating the standard deviation. When *E. coli* cells were stained with the anti-*E. coli* antibodies, the detected bacteria number is 1.5×10^6 cell ml^{-1}. By contrast, when *E. coli* cells were stained with the anti-*S. flexneri* antibodies, the detected bacteria number is 9×10^4 cell ml^{-1}. The difference of the detected bacteria number shows much higher binding efficiency of the interaction between *E. coli* and anti-*E. coli* antibodies, compared to that between *E. coli* and anti-*S. flexneri* antibodies. Similarly, *S. flexneri* cells bound to anti-*S. flexneri* antibodies with higher binding efficiency than anti-*E. coli* antibodies.

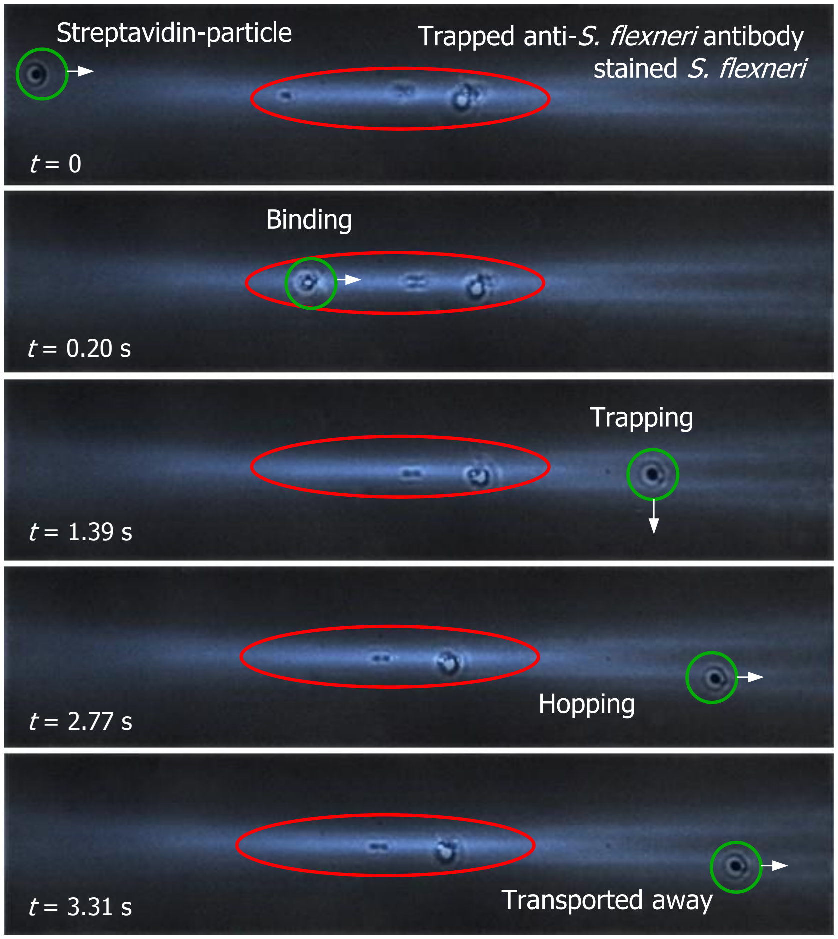
Streptavidin-particle
Trapped anti-*S. flexneri* antibody stained *S. flexneri*
t = 0
Binding
t = 0.20 s
Trapping
t = 1.39 s
Hopping
t = 2.77 s
Transported away
t = 3.31 s

(a)

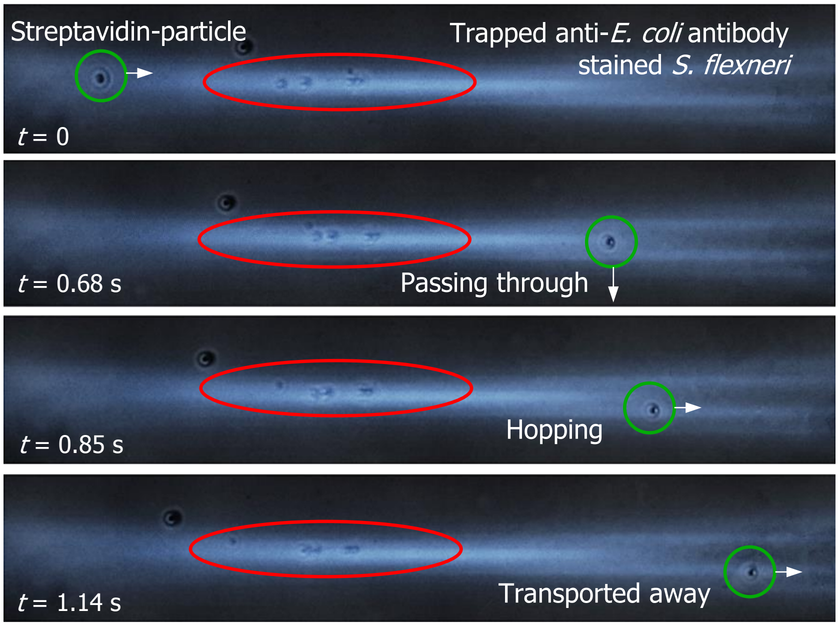
Streptavidin-particle
Trapped anti-*E. coli* antibody stained *S. flexneri*
t = 0
Passing through
t = 0.68 s
Hopping
t = 0.85 s
Transported away
t = 1.14 s

(b)

Supplementary Fig. 11 | Experimental demonstration of selective binding and hopping of *S. flexneri*. Experimental observation of the streptavidin-conjugated microparticle (a) hopping with anti-*S. flexneri* antibody stained *S. flexneri*, (b) passing through anti-*E. coli* antibody stained *S. flexneri*. The laser power was 400 mW and flow velocity was 50 μm s^{-1}.

Supplementary Table 1 | Mean residence time for different bacteria and antibodies (Sample size: 300 events).

Condition	Mean residence time (s)
E. coli	0.09 ± 0.03
E. coli + anti-*E. coli* antibody	0.05 ± 0.02
E. coli + anti-*S. flexneri* antibody	0.11 ± 0.08
S. flexneri + anti-*S. flexneri* antibody	0.11 ± 0.10
S. flexneri + anti-*E. coli* antibody	0.12 ± 0.10

Supplementary Note 1 | The role of non-uniformity of the intensity between the four hotspots in relationship to the three hopping mechanisms

For hopping induced by particle bypassing, the pre-trapped particle blocks the upcoming particle and causes it to be trapped on the right side of the hotspot 1. Since the strength of the potential well in hotspot 2 or 4 is much stronger than that at the right edge of hotspot 1, the particle will eventually hop to hotspot 2 or 4 and the residence time of the particle in hotspot 1 is determined by the intensity of hotspot 2 or 4.

For hopping induced by particle collision, the pre-trapped particle is directly pushed to hotspot 2 or 4 and the lateral distance (1.4 μm) between the two potential wells is the key factor inducing the hopping event. If the distance is too long, the pre-trapped particle may not be pushed to hotspot 2 or 4 from hotspot 1. On the contrary, if the distance is shorter, particle hopping will be easier.

For hopping induced by particle aggregation, both the lateral distance and the relative intensity of potential wells matter. The intensity of hotspot 2 or 4 will change the critical angle of the hopping since the particle has crossed the safe barrier as shown in Figure 4c.
