# Supplementary material for: Sculpting nanoparticle dynamics for single-bacteria-level screening and direct binding-efficiency measurement
Source: Nat Commun. 2018 Feb 26;9:815. doi: 10.1038/s41467-018-03156-5 (PMC5827716; doi:10.1038/s41467-018-03156-5)
Supplement: Supplementary file 3 — Description of Additional Supplementary Files [file 41467_2018_3156_MOESM3_ESM.pdf]

Description of Additional Supplementary Files

File Name: Supplementary Movie 1
Description: Particle bypassing induced hopping. The incoming particle passed through the pre-trapped particle and was trapped in the extra trapping position induced by the focusing of light by the pre-trapped particle. The incoming particle later hopped to the adjacent potential well due to the shallow potential well. The Movie is also available on Youtube with the link: https://youtu.be/tvUtKLRwH20.

File Name: Supplementary Movie 2
Description: Snookering-like collision induced hopping. The incoming particle had an on-side collision with the pre-trapped particle. The collision pushed the pre-trapped particle away from the previous potential well to the adjacent potential well due to the momentum transfer. The Movie is also available on Youtube with the link: https://youtu.be/H7qBSYRDNKY.

File Name: Supplementary Movie 3
Description: Particle aggregation induced hopping. The aggregation of three particles made the trapping of outside particles in the conjugation unstable. The outside two particles eventually hopped away as a particle pair, leaving only one particle stably trapped in the potential well. The Movie is also available on Youtube with the link: https://youtu.be/V_tl4c3_M-0.

File Name: Supplementary Movie 4
Description: Antibody selection with *E. coli* cells. The *E. coli* that stained with anti-*E. coli* antibody bound and hopped away with the particle. While the *E. coli* that stained with anti-*S. flexneri* antibody remained trapped in the potential well. The Movie is also available on Youtube with the link: https://youtu.be/i1ANsvxA_S8.

File Name: Supplementary Movie 5
Description: Antibody selection with *S. flexneri* cells. The *S. flexneri* that stained with anti-*S. flexneri* antibody bound and hopped away with the particle. While, the *S. flexneri* that stained with anti-*E. coli* antibody remained trapped in the potential well. The Movie is also available on Youtube with the link: https://youtu.be/sQlP19SXayM.

File Name: Supplementary Movie 6
Description: Drag force induced hopping. Control of flow velocity to enable the hopping back of particle from Hotspot 3 to 4. The Movie is also available on Youtube with the link: https://youtu.be/nX8m6iiMFKY.

File Name: Supplementary Movie 7
Description: Controllable particle hopping loop in the optofluidic lattice. The Movie is also available on Youtube with the link: https://youtu.be/X6dh4a8DhQc.
